# Folie a famille

**DOI:** 10.4103/0019-5545.58899

**Published:** 2010

**Authors:** Ashish Srivastava, H. A. Borkar

**Affiliations:** Department of Psychiatry, Institute of Psychiatry and Human Behavior, Bambolim, Goa - 403 202, India

**Keywords:** Folie a famille, folie imposee, paranoid schizophrenia, shared psychotic disorder

## Abstract

Shared psychotic disorder is often read as case report but not studied in length and rarely looked at in common clinical practice in psychiatry. Only a small percentage of cases involve families. Folie a famille is characterized as a shared psychotic disorder within a family in more than two members. The involved patients have an unusually close relationship and are isolated from others. We describe here a case of folie a famille involving a nuclear family consisting of the husband, the wife, and their three children. The primary patient was suffering from paranoid schizophrenia with prominent delusions of persecution that were imposed upon and later shared by his family. Temporary separation decreased the intensity of shared delusions in the other family members.

## INTRODUCTION

Shared psychotic disorder (SPD) was first described by Jules Baillarger in 1860, who termed this condition as “folie a communiqué”. It has been variously called as psychosis of association, shared paranoid disorder, communicated insanity, contagious insanity, folie a deux, folie a trios, folie a quatre, folie a cinq and folie a famille.[1,2] Lasegue and Falret coined the term “folie a deux” or ‘insanity or psychosis of two’ in their classic paper titled ‘Lafolie a deux’ in 1877.[3] Folie a famille is said to be present when more than two members of the same family are involved. It is classified as SPD in Diagnostic and Statistical Manual of Mental Disorders (DSM) 4^th^ edition[4] and induced delusional disorder in International Classification of Diseases (ICD) 10^th^ edition.[5]

SPD is said to be rare[6,7] and SPD involving the entire family is even more rare.[1] Its true population prevalence is difficult to assess.[8] The available literature on this condition is scant and limits mainly to case reports. Gralnick in 1942, in his excellent review, defines it as “the transfer of delusional ideas and/or abnormal behavior from one person to another or one person to several others, related or unrelated, who have been in close association with the primary affected person.”[9] The essential theme of SPD is often persecutory or grandiose. The delusions are first manifested in the dominant personality, who in turn influences the weaker personalities and suggestible and less intelligent people. It is identified more frequently in women,[10,11] reflecting the traditional submissive role of females in the family. The involved cases have an unusually close relationship and are isolated from others by language, culture, or geography; most of the reported relationships have been within the nuclear family.[12]

We describe here a case of folie a famille involving a nuclear family consisting of the husband (40 years), wife (35 years), and their three children (14, 10 and 6 years old). It may be the first reported case in our region.

## CASE REPORT

V.F (40-year-old male) was brought to our hospital by police with a reception order from the court after a complaint was made to the police by his sister and his neighbors. V.F was diagnosed to be suffering from paranoid schizophrenia for last 4 years for which he had received no treatment. He believed that people in his locality were his enemies and would kill him and his family. He had been unemployed and was at home for the last 4 years. On further evaluation, he was also diagnosed as suffering from anti-social personality disorder along with alcohol abuse. He stopped his wife and children from going out of the house. He stopped his children from going to school. He would guard, whole day, the gate of his house. Over the last 1 year, his wife and children also started believing that the people in their locality wanted to kill them and stopped any kind communication with them. The delusional context was same in all involved family members. The wife and the children had no past history of psychiatric illness but showed dependent personality traits. V.F had family history of psychiatric illness in his mother, details of which could not be ascertained. He was not suffering from any medical illness.

All the family members were intimately associated; the family showed growing social isolation from the social network of neighbors and relatives. None of the family members were employed. The onset of symptoms in all the other family members was 3 years after the symptoms in the primary patient, V.F, started. The primary patient demonstrated most of the other criteria for schizophrenia according to DSM-IV, while other family members showed most features of shared psychotic disorder.

V.F. was started on antipsychotic medication, tablet Risperidone 2 mg twice per day, which was increased to 6 mg/day in two divided doses, along with electroconvulsive therapy. The wife and children visited the patient only twice during the course of his admission in the hospital which lasted around 2 months. They refused any kind of treatment but their delusional beliefs weakened over the 2 months period. The patient improved and was discharged on tablet Risperidone 6 mg/day, as the family did not want to further keep him in the hospital. Following discharge from the hospital, the family refused to live separately from the patient. The patient did not comply with treatment and was lost to follow up after four outpatient visits.

## DISCUSSION

This case illustrates a case of folie a famille involving a couple and their three children. In the above family, V.F was the dominant person who was suffering from schizophrenia and had prominent delusion of persecution while other members of the family received a diagnosis of SPD. When V.F prevented his family from having contact with neighbors, friends, and extended family, his delusional beliefs spread to involve the other members of his family viz. his wife and children. His wife and children readily identified with and depended on him. The patient was imposing his ideas on his family, causing a considerable disruption in their lives and due to his delusional beliefs they were isolated from the rest of the community. The family passively submitted to and shared the imposed delusional system of the patient. They did not have any other primary psychiatric diagnosis. They lacked insight and refused treatment, as is the case with most patients of SPD. With temporary separation, delusional beliefs of family decreased in intensity. The case reflects the folie imposee subtype of SPD among the four subtypes, impose, simultanee, communiqué and induite, described by Gralnick in 1942.

The progression of delusional symptoms to a folie a famille is thought to reflect an attempt of a family to maintain cohesiveness in the presence of a perceived hostile environment.[13] Sharing these delusions in the family contributes to the development of a delusional “psuedocommunity”. It is the most impressive illustration of a pathological relationship.[14] Predisposing factors in our case were social isolation and dependent personality traits in the family members. It is generally accepted that a dyad composed of charismatic psychotic inducer and an induced person with dependent traits is necessary for the development of SPD.[4]

The above report demonstrates the complexity of folie a famille and the severity of behavioral consequences and psychosocial impairments caused by induced or shared delusions. As the literature shows, the disorder is rare, but proper recognition of this disorder can result in successful treatment outcomes by separation of patients and psychopharmacological treatment. The problem of compliance with treatment poses an obstacle, as it happened later in our case.
